# Efficacy and safety of Sangbaipi Decoction in patients with acute exacerbation of chronic obstructive pulmonary disease: A protocol for systematic review and meta analysis

**DOI:** 10.1097/MD.0000000000022917

**Published:** 2020-10-30

**Authors:** Jiazhou Li, Junlin Jiang, Chunxiang Jing, Wenjiang Zheng, Huashan Pan

**Affiliations:** a School of sport and health of Guangzhou University of Chinese Medicine; b The First Clinical Medical School of Guangzhou University of Chinese Medicine; c Department of Science and Technology of Guangdong Food and Drug Vocational College, Guangzhou, Guangdong Province, China.

**Keywords:** meta-analysis, protocol, systematic review, acute exacerbation of chronic obstructive pulmonary disease, Sangbaipi decoction

## Abstract

**Background:**

Chinese medicine Sangbaipi decoction is extensively applied to the therapy of acute exacerbation of chronic obstructive pulmonary disease (AECOPD) in China. However, owing to the low quality, small sample size, and methodological heterogeneity of these studies, this conclusion is not convincing. Consequently, it is necessary to systematically evaluate the clinical efficacy and safety of Sangbaipi Decoction in the treatment of AECOPD patients, and provide high-quality evidence for its clinical application.

**Methods:**

We will follow the preferred reporting items for systematic review and meta-analysis (PRISMA) for reporting the results of the review in this study. We will utilize the Review Manage software V5.3.0 (The Nordic Cochrane Center, The Cochrane Collaboration, 2014, Copenhagen, Denmark) to assess the risk of bias and visualize the results. We will use Stata software (version 15.0, StataCorp, College Station, TX) to perform the meta-analysis.

**Ethics and dissemination:**

This study is a systematic review and meta-analysis protocol of Sangbaipi decoction on AECOPD, participants were not recruited and data were not collected from participants, so ethical ratification is not required.

**Results:**

This study will provide high-quality synthesis of the effectiveness and safety of Sangbaipi decoction for AECOPD. Upon completion, the results will be submitted to a peer-reviewed journal.

**Conclusion:**

The efficacy and safety assessment of Sangbaipi decoction for AECOPD will be supported by this protocol.

**Registration number:**

PROSPERO CRD 42019138405.

## 1 Introduction

Chronic obstructive pulmonary disease (COPD) describes a predominantly smoking-induced small airway and/or emphysematous disease associated with airflow limitation.^[1]^ Considered progressive, irreversible, and responsible for substantial morbidity and mortality worldwide, the number of patients with COPD in China has reached 100 million.^[2]^ Acute exacerbation of chronic obstructive pulmonary disease (AECOPD) is defined as an acute worsening of respiratory symptoms that result in additional therapy, which is a serious event in the management of COPD. Therefore, AECOPD remains the subject of vigorous study.

At present, the conventional Western medicine treatment options for AECOPD include anti-infection, anti-inflammatory and anti-asthmatic, bronchial expansion, and mechanical ventilation therapy.^[3]^ The application of glucocorticoids can benefit patients, but many studies have shown that glucocorticoids are not effective in the treatment of COPD and the development of airway inflammation, which also cannot reverse the decline in lung function. There is a phenomenon of glucocorticoid insensitivity in AECOPD.^[4]^ Therefore, the clinical efficacy of AECOPD needs to be improved.

In recent years, clinical studies have also shown that Sangbaipi Decoction combined with conventional Western medicine treatment of AECOPD can improve the clinical efficacy,^[5]^ but there are some limitations, such as single reports, scattered research, small sample size. With the development of evidence-based medicine, it has opened up new research fields in the clinical efficacy evaluation of Chinese medicine, produced new research ideas and methods, and promoted the progress of clinical research in Chinese medicine.^[6,7]^ Therefore, to systematically review the efficacy and safety of Sangbaipi decoction in treating AECOPD may provide higher quality evidence for further clinical application.

## 2 Methods

### 2.1 Protocol registration

The preferred reporting items for systematic review and meta-analysis (PRISMA) for reporting the results of the review will be followed by this research.^[8]^ We have registered this plan in the International prospective register of systematic reviews (PROSPERO) and the number is CRD 42019138405. If there are any adjustments throughout the study, we will fix and update the details in the final report.

### 2.2 Eligibility criteria

We will use the PICOS (participant-intervention-comparative-outcome-study design) framework for the eligibility criteria of our review as Table 1.

**Table 1:**
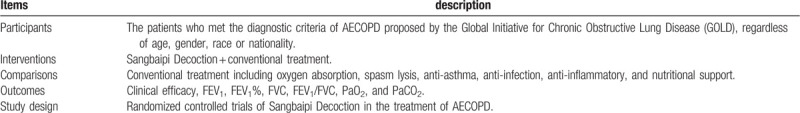
The eligibility criteria of our review.

### 2.3 Search strategy

We will search each database from the built-in until August 2020. The English literature searches PubMed, Cochrane Library, EMBASE, and Web of Science, while the Chinese literature comes from CNKI, SinoMed, VIP, and Wangfang database. What is more, targeted grey literature searches will be conducted against the Clinical trials.gov and the international Clinical trial registration platform retrieval portal to identify ongoing and completed trials. In all database searches, there will be no restrictions on languages. However, once the search has been conducted, papers that are not in English or Chinese will be excluded. The search strategy of PubMed is as Table 2:

**Table 2:**
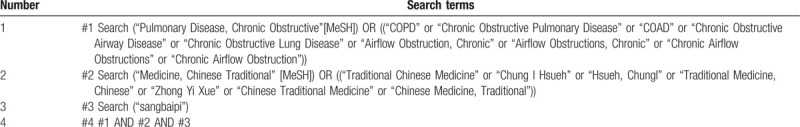
Search strategy for the PubMed database.

### 2.4 Study selection and data extraction

**Figure 1:**
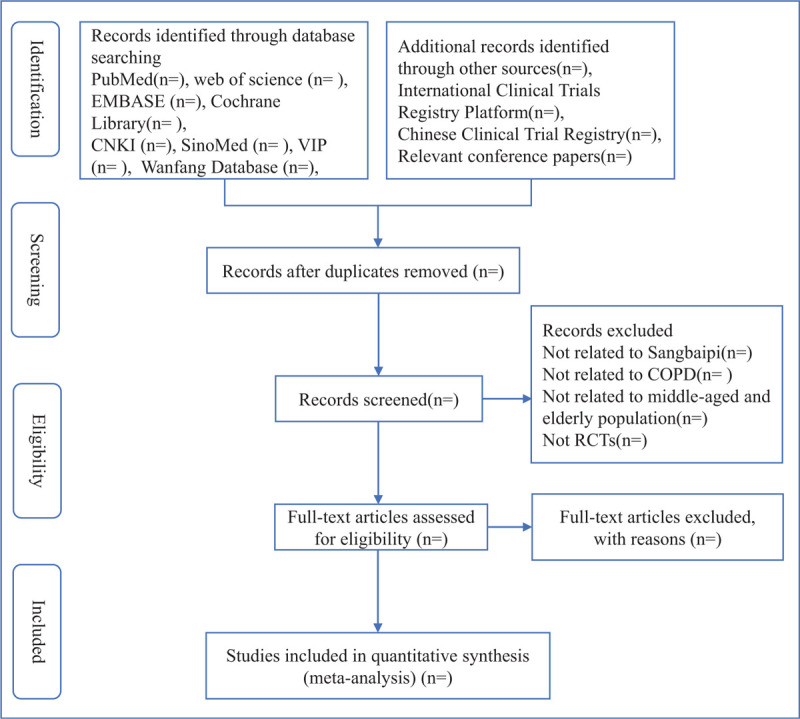
Flow diagram of study selection process.

We will manage the electronic citations downloaded from 8 databases, then they will be imported into EndNote X7.0. Once duplicates have been removed, 2 reviewers will retrieve all the literature independently. The reviewers will screen titles and abstracts, excluding nonrelevant papers according to the inclusion/exclusion criteria. If there is any disagreement in the process of document inclusion, it will be decided through discussion. We will use Microsoft Excel 2019 to collect relevant information and extract data from it. The extracted information will be classified into 5 parts as Table 3. The screening process will be presented with reference to the PRISMA statement as Figure 1.

**Table 3:**
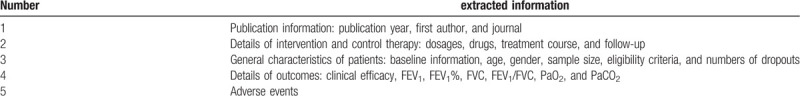
Basic information of included studies.

### 2.5 Quality assessment/risk of bias assessment

All the included studies will be evaluated in accordance with the Risk of Bias Tool of guidelines of the Cochrane Handbook for Systematic Reviews of interventions.^[9]^ Two review authors will evaluate the original design independently. Bias risk through 7 assessment trials: random sequence generation (selection bias), allocation concealment (selection bias), blinding of participants and personnel (performance bias), blinding of outcome assessment (detection bias), incomplete outcome data (attrition bias), selective reporting (reporting bias), and other bias. Each item is classified as “Low risk", “High risk” or “Unclear risk”, and the percentages for each category in each source of bias analyzed will be described by RveMan 5.3.0 software and the results interpreted taking the risk of bias into account. The disagreement of bias risk will be resolved through further discussion with a third independent reviewer. Sensitivity analysis will also be conducted to exclude the studies reporting the high risk of bias in any domain analyzed. We will future assess the certainty of the evidence of the primary outcome through the “grading of recommendations assessment, development, and evaluation” (GRADE) system.^[10]^ Based on 5 areas (bias, indirection, inconsistency, imprecision, and risk of publication bias), the quality of evidence is divided into one of 4 levels: high, medium, low, and very low. The GRADE profiler 3.2 will be employed for analysis.

### 2.6 Statistical analysis

We will use Stata 15 software (version 15.0, Stata Corp, College Station, TX) to complete the meta-analysis. Specifically as follows: The confidence interval (CI) for continuous and dichotomous variables will be set to 95%. For dichotomous variables, we will apply the odds ratio (OR) to analyze. For continuous data, the mean difference (MD) or standardized MD (SMD) with 95% CI will be estimated. For continuous variables with the same measurement unit and little difference in means, we will use the WMD, and for continuous variables with different measurement units and large differences in means, we will use the SMD. Heterogeneity analysis will be calculated based on the *X*^2^ test, and the degree of heterogeneity will be determined by the *I*^2^ values (*I*^2^ > 50% or not) or *P* value (*P* < .05 or not).^[11]^ If *I*^2^ < 50% and *P* > .05, the meta-analysis will be performed using the Mantel–Haenszel fixed-model. If *I*^2^ ≥ 50% and *P* < .05, we will use random-effects models for the meta-analysis. We will use subgroup, meta regression analyses, and sensitivity analyses to explore the sources of heterogeneity.^[12]^ If the source of heterogeneity cannot be known, the synthetic analysis will be abandoned and descriptive analysis will be adopted. Descriptive statistical analysis will be performed on adverse reactions. For indicators with a number of studies ≥10, we will draw a funnel chart to determine whether the included studies have publication bias,^[13]^ and use Harbord weighted linear regression to quantitatively test the publication bias. When *P* < .05, it is considered that there is a significant publication bias.

### 2.7 Ethics and dissemination

In consideration of the systematic review of this protocol, ethical ratification is not required. In this study, participants were not recruited and data were not collected from participants. The review will be disseminated through peer-reviewed publications.

## 3 Discussion

COPD is a common, preventable and treatable disease that is characterized by persistent respiratory symptoms and airflow limitation that is due to airway and/or alveolar abnormalities usually caused by significant exposure to noxious particles or gases. The chronic airflow limitation that is characteristic of COPD is caused by a mixture of small airways disease and parenchymal destruction, the relative contributions of which vary from person to person.^[14]^ Sangbaipi decoction is extensively applied to the therapy of AECOPD in China. However, owing to the low quality, small sample size, and methodological heterogeneity of these studies, this conclusion is not convincing. Consequently, it is necessary to systematically evaluate the clinical efficacy and safety of Sangbaipi Decoction in the treatment of AECOPD patients, and provide high-quality evidence for its clinical application.

Sangbaipi decoction is composed of Mori Cortex (Sangbaipi), Arum Ternatum Thunb (Banxia), Perillae Fructus (Zisuzi), Amygdalus Communis Vas (Kuxingren), Fritillariae Thunbrgii Bulbus (Zhebeimu), Scutellariae Radix (Huangqin), Coptidis Rhizoma (Huanglian), and Gardeniae Fructus (Zhizi), and has the effect of clearing away heat and reducing phlegm. Pharmacological researches show that Sangbaipi has anti-inflammatory effects^[15]^ and immunomodulating activity,^[16]^ Banxia has protective effects against allergic airway inflammation,^[17]^ its treatment protected the airway from ICS withdrawal-induced mucus hypersecretion and airway inflammation by inhibiting ERK activation,^[18]^ Zisuzi has anti-inflammatory and immune-regulating pharmacological effects.^[19]^ Huangqin possesses anti-inflammatory functions by inhibiting hyperpermeability, expression of CAMs, and adhesion and migration of leukocytes.^[20]^ Evidence of the efficacy and safety of Sangbaipi decoction for AECOPD will be provided by this study, it will be able to assist clinicians in making Sangbaipi decoction-related decisions and will be beneficial to patients with AECOPD in the world. The design of this protocol conforms to the guideline of Preferred reporting items for systematic review and meta-analysis protocols (PRISMA-P) 2015.^[21]^ After completion, the results of this systematic review and meta-analysis will be submitted to a peer-reviewed journal.

## Author contributions

**Conceptualization:** Jiazhou Li, Huashan Pan.

**Data curation:** Wenjiang Zheng.

**Funding acquisition:** Chunxiang Jing, Huashan Pan.

**Investigation:** Wenjiang Zheng.

**Methodology:** Jiazhou Li, Junlin Jiang.

**Project administration:** Huashan Pan.

**Software:** Jiazhou Li, Junlin Jiang.

**Supervision:** Chunxiang Jing.

**Validation:** Jiazhou Li, Junlin Jiang.

**Writing – original draft:** Jiazhou Li, Junlin Jiang.

**Writing – review & editing:** Huashan Pan.
